# Prognostic and clinicopathological significance of survivin in gynecological cancer

**DOI:** 10.3389/or.2024.1444008

**Published:** 2024-12-02

**Authors:** Agapiti H. Chuwa, David H. Mvunta

**Affiliations:** ^1^ Department of Physiology, Mbeya College of Health and Allied Sciences, University of Dar es Salaam, Mbeya, Tanzania; ^2^ Department of Obstetrics and Gynecology, Muhimbili University of Health and Allied Sciences, Dar es Salaam, Tanzania; ^3^ Department of Surgical Oncology, Ocean Road Cancer Institute, Dar es Salaam, Tanzania

**Keywords:** survivin, BIRC5, gynecological cancer, chemoresistance, targeted therapy

## Abstract

Survivin belongs to the inhibitor of apoptosis protein (IAP) family and is encoded by the baculoviral inhibitor of apoptosis repeat-containing, or BIRC5, gene. It is preferentially expressed in cancers with functional complexity in cell signaling cascades such as extracellular signal-regulated kinases (ERK), mitogen-activated protein kinases (MAPK), heat shock protein-90 (HSP90), epidermal growth factor receptor (EGFR), phosphoinositide 3-kinase (PI3K), signal transducer and activator of transcription (STAT), hypoxia-inducible factor-1 alpha (HIF-1α), vascular endothelial growth factor (VEGF), and others. Survivin plays a role in cell division and cell death, properties that have attracted a large body of research to decipher its therapeutic and prognostic significance in cancer. Survivin has tumor-promoting effects in endometrial (EC) and ovarian (OC) cancers, and its upregulation in endometrial cancer has been associated with poor overall survival (OS). While survivin protein is abundantly expressed in OC, it is barely detectable in normal ovarian tissue or benign ovarian tumors. Survivin expression is also a marker for cervical intraepithelial neoplasia (CIN) and high-risk human papillomavirus, and a predictor of viral clearance and prognosis in uterine cervical cancer (UCC). Furthermore, nuclear survivin expression is very low in normal vulvar squamous epithelium and increases to become abundant in vulvar invasive squamous cell carcinoma (ISCC), conferring resistance to apoptosis in vulvar carcinogenesis. In this review, we discuss in detail the impact of survivin signaling on gynecological cancers and provide insight on its therapeutic and diagnostic potential, existing research gaps, and areas for future research.

## 1 Introduction

**FIGURE 1:**
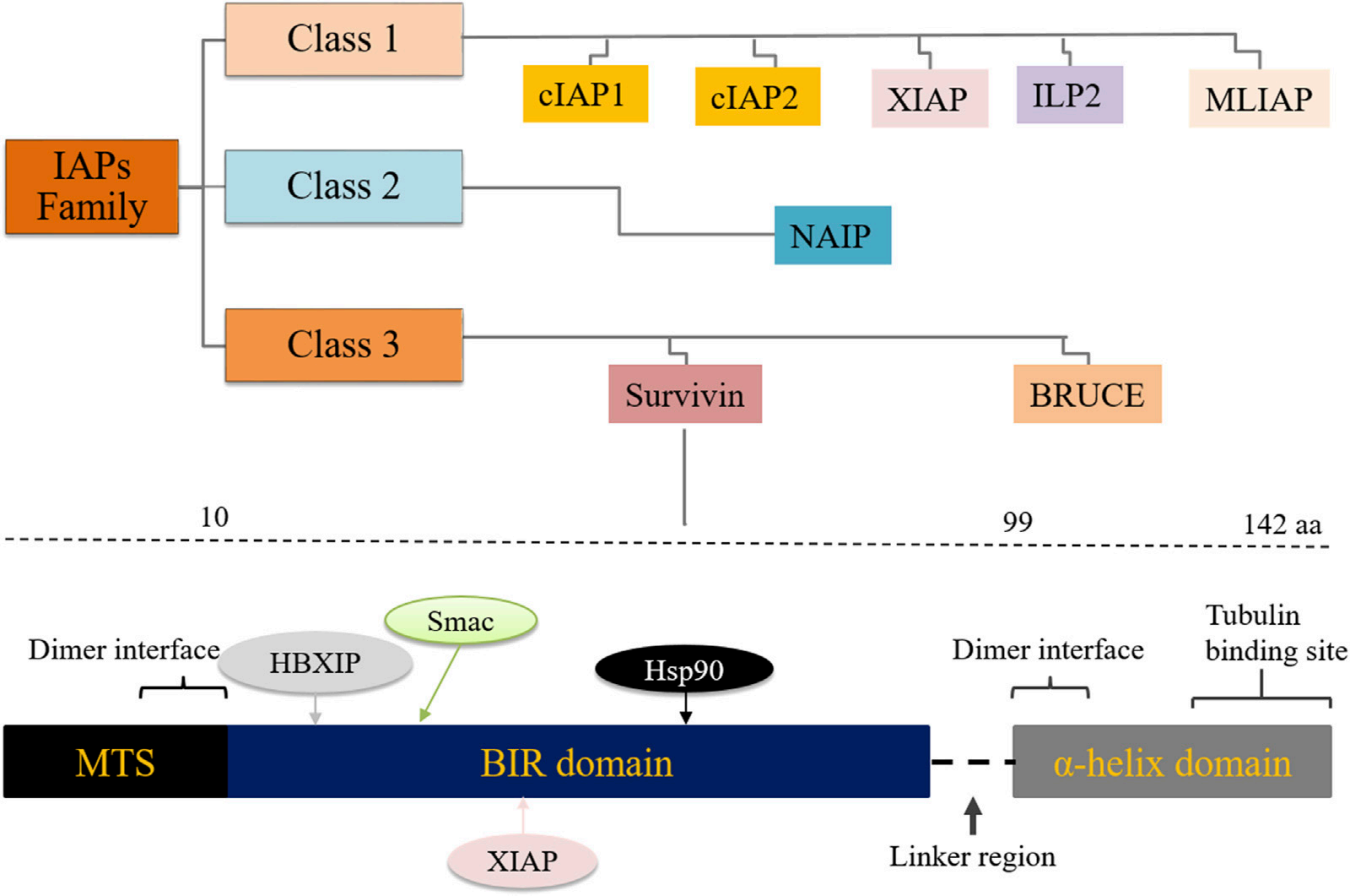
Classification of the inhibitor of apoptosis (IAP) protein family and the survivin gene BIRC5 showing interacting partner proteins/molecules. MTS: mitochondrial targeting sequence, HBXIP: hepatitis B X-interacting protein.

Survivin, also known as BIRC5, is the smallest member of the inhibitor of apoptosis proteins (IAPs) family, which plays a role in mitotic regulation and inhibition of apoptosis as well as carcinogenesis (1). Eight members of the IAPs have been described namely, X-linked inhibitor of apoptosis (XIAP), NLR family apoptosis inhibitory protein (NAIP), cellular inhibitor of apoptosis proteins-1 and -2 (cIAP1, cIAP2), IAP-like protein-2 (ILP2), melanoma inhibitor of apoptosis (MLIAP) also known as livin’, survivin and baculovirus inhibitor of apoptosis repeat-containing ubiquitin conjugating enzyme (BRUCE). Human survivin has 142 amino acids and is a 16.5 kDa protein. The survivin gene was first cloned by Ambrosini et al (2). The BIRC5 gene spans 147 kb and consists of three introns and four exons. The survivin gene has two functional domains; a BIR (baculoviral IAP repeat-containing) domain in the N-terminus and an α-helix in the C-terminus (Figure 1).

Both domains control the regulation of cell mitosis, but only the BIR domain is involved in the regulation of apoptosis. BIRC5 lacks the caspase activation and recruitment domain (CARD) motif. This feature prevents it from directly binding to and inhibiting caspases. It also lacks the IAP C-terminal really interesting new gene (RING) finger domain, which would bind zinc ions with the BIR domain (3). While the helical domain of the BIRC5 gene interacts mainly with tubulin structures, (Figure 1), the BIR domain plays a central role in the control of cell division and cell death (4, 5). Several genetic survivin splice variants (isoforms) with unique functions have been documented, including survivin-wt, survivin-2α, survivin-2B, survivin-3B and survivin-ΔEx-3 (6, 7). Survivin is expressed in proliferating cells, including cancer stem cells (CSCs), and is barely detectable in well-differentiated somatic cells. It can be detected in virtually all cellular compartments, including the extracellular matrix, the outer surface of the cell membrane, the cytoplasm, mitochondria, exosomes and the nucleus. Survivin plays a central role in several cellular pathways including cell division, angiogenesis, apoptosis and carcinogenesis (8). Survivin functions are influenced by its extensive post-translational modification, reversible dimerization and subcellular localization (9). Overexpression of survivin protein has been documented in a variety of human cancers, including pancreatic, breast, gastric, lung, uterine, ovarian, esophageal and cervical cancers (10, 11, 12, 13).

## 2 Survivin signaling and gynecological cancer

### 2.1 Survivin signaling in endometrial cancer

Among gynecological cancers, survivin signaling has been best studied in endometrial cancer. Although survivin expression has been reported in non-malignant endometrium, the levels are comparatively low. Information on the precise function of survivin in non-malignant endometrial tissue is limited; however, it is overexpressed in cycling endometrium, early pregnancy placenta, endometriosis and endometrial hyperplasia (14, 15, 16, 17, 18, 19, 20). There are conflicting reports on the role of survivin in endometrial physiology. Garcia et al. studied normal/low abortion (CBA/J x BALB/c) and abortion-prone (CBA/J x DBA/2J) mouse models and showed that high levels of survivin expression may be associated with pregnancy loss in mice (21). In contrast, work by Fest et al. using the same mouse models showed decreased levels of survivin mRNA and protein expression in the abortion-prone mice compared to the normal group, suggesting that survivin plays a role in promoting trophoblast cell survival and proliferation, thus maintaining pregnancy (22). It has also been shown that BIRC5 gene expression is significantly repressed in the deciduae of interleukin-11 receptor alpha null (IL-11Rα) mice, which are infertile due to defective decasualization and impaired trophoblast invasion (23). Interestingly, treatment of cells with IL-11 stimulated survivin expression, suggesting that survivin may be a target of IL-11 in the decidua and may play a role in maintaining fertility. IL-11Rα has been implicated in doxorubicin chemoresistance in high-grade endometrioid adenocarcinoma, the most common type of endometrial cancer (24). Overexpression of survivin in endometrial cancer has been documented and correlates with both tumor stage and grade; some of the reports are shown in Table 1.

**TABLE 1:** Survivin protein expression expression and its correlated effects in gynecological cancers.

Study type	Cancer type	Sample	Effect	Reference
Experimental	Endometrial cancer	Tissue samples: 99 patients	Overexpressed in 88% Correlates with grade, clinical stage, and invasiveness	(25)
Experimental	Endometrial cancer	Cell lines: 16 cancer cell lines gene analysis	Overexpressed in 87.5%.; Marker of poor prognosis	(26)
Experimental	Ovarian cancer	Tissue samples: 49	Overexpressed in 74% Correlates with poor prognosis	(27)
Meta-analysis	Ovarian cancer	2,233 patients	Associated with clinical stage, grade, clinical outcome, and survival Marker of poor prognosis	(28)
Experimental, gene analysis	Vulvar cancer	55 VSCC and 50 normal vulvar sections	Strongly correlated with clinical stage	(29)
Experimental	Vulvar cancer	Tissue samples	Strongly correlated with higher tumor grade	(30)
Experimental: *In vitro* and *in vivo*	Cervical cancer	Cancer cell lines, xenografted tumors	Causes resistance to radiotherapy	(31)

Stavropoulos A. et al. examined survivin expression in primary endometrial cancer tissue samples and found that 88% of the samples (n = 99) were immunopositive. Of the samples examined, 60% showed co-expression of survivin, phosphatase and tension homolog (PTEN) and p53 (25). The sum of survivin staining intensity and scores was significantly associated with patient age, histological grade, clinical stage and metastasis. In our previous work published in *Gynecologic Oncology*, we also showed that survivin was expressed in 87.5% of endometrial cancer cell lines and that high expression of BIRC5 gene was significantly associated with poor progression-free survival (*p* = 0.006), and was an independent prognostic factor (HR = 1.97, 95% CI = 1.29–4.5, *p* = 0.045) (26). Other reports have also shown a positive correlation between high levels of survivin expression and advanced clinical stage, histological grade, invasiveness and prognosis of endometrial cancer (20), (32, 33, 34). Survivin is involved in several cellular signaling pathways in endometrial cancer. The survivin gene BIRC5, among other hub genes (GTSE1, AURKA and KNSTRN), has been associated with AKT1, one of three closely related serine/threonine protein kinases that regulate various cellular processes including metabolism, growth, proliferation, angiogenesis and cell survival (35).

PTEN is an established tumor suppressor gene encoding a phosphatase; a key regulatory enzyme in signaling via the PI3K-AKT-mTOR pathway which controls cell growth, mitosis, metabolism and apoptosis. PTEN functions by antagonizing the PI3K/AKT pathway through dephosphorylation of PIP3, which results in decreased translocation of AKT to cell membranes, subsequently downregulating its phosphorylation and activation (36). In endometrioid endometrial cancer (which accounts for 80% of endometrial cancers), molecular aberrations in this pathway occur in approximately 80%–90% of cases (37, 38, 39). Guha M. et al. investigated the association between survivin expression, PTEN and p53 and confirmed that PTEN-mediated endogenous tumor suppression involves silencing of the survivin gene, BIRC5 (40). This association may be a potential therapeutic target, as PTEN has been confirmed as a major target gene in endometrial carcinogenesis and a predictive marker for the development of high-grade endometrial cancer (41, 42). Furthermore, hypermethylation of the BIRC5 promoter in endometrial cancer is known to block the binding of TP53 to its promoter region, thereby increasing survivin expression (43). The BIRC5 promoter is unmethylated in normal endometrial tissue, but methylation levels increase from low to high grade endometrial cancer and correlate with increased survivin protein expression (44).

The tumor suppressor gene TP53 and the transcription factor Egr1 are known repressors of the survivin gene promoter. The putative p53-mediated inhibition of the survivin gene BIRC5 has been demonstrated (45). In addition, p53 suppresses tumor development by activating apoptosis signaling genes such as PUMA, BAX and Noxa to induce the process of programmed cell death (apoptosis) in the event of irreparable DNA damage. The frequency of TP53 mutations is 10%–40% and approximately 90% in type I and type II endometrial cancer, respectively. Several studies have investigated p53 protein expression levels and their significance in endometrial cancer (46, 47, 48, 49, 50, 51). Nuclear accumulation of p53 protein resulting from a missense mutation in the TP53 gene is observed as overexpression by immunohistochemistry (IHC). High p53-positive expression was observed in 10%–44% of type I endometrial cancer and 30%–86% of type II endometrial cancer. Thus, the association between survivin and the two tumor suppressor genes, PTEN and TP53, is likely to be an attractive molecular therapeutic target, as mutation of these two genes is the most common gene mutation documented in endometrial cancer (52, 53, 54). In addition, co-expression of survivin and vascular endothelial growth factor (VEGF), a highly specific vascular endothelial cell mitogen that promotes angiogenesis in tumor tissue, has recently been found to be associated with recurrence-free progression and overall survival in endometrial cancer patients (55, 56, 57). Such co-overexpression is associated with the International Federation of Gynecology and Obstetrics (FIGO) stage, deep myometrial invasion, lymph node metastasis and survival status, and was an independent predictor of poor prognosis (58).

Progestin therapy is a conservative treatment often used in younger endometrial cancer patients who are not candidates for surgery or who wish to preserve fertility, but about 30% of patients do not respond to progestin therapy. Survivin signaling has been strongly implicated in hormone therapy resistance in endometrial cancer. Overexpression of survivin and nuclear factor erythroid 2-related factor-2 (Nrf2) has been reported in partially progestin-responsive and progestin-resistant EC tissue samples, and negative expression of these biomarkers in responsive tissue samples (59). Survivin-induced progestin resistance was indeed confirmed by pre- and post-treatment assays showing a 20-fold decrease in survivin expression in progestin responders and a non-significant change in survivin expression in non-responders (20). We have also previously reported that treatment of endometrial cancer cells with estradiol significantly induced co-expression of nuclear ERα and survivin proteins (*p* < 0.001), whereas inhibition of survivin caused apoptotic cell death (60). Endometrial cancer tends to progress slowly and is often diagnosed at an early stage due to noticeable symptoms such as abnormal vaginal bleeding. This provides an opportunity for early intervention. In contrast, ovarian cancer is often called the ‘silent killer’ because it tends to progress quickly and is often diagnosed at an advanced stage due to subtle and non-specific symptoms such as bloating, pelvic pain or gastrointestinal problems.

### 2.2 Survivin signaling in ovarian cancer

Ovarian cancer is associated with the highest mortality of all gynecological tumors. Among malignant ovarian tumors, epithelial ovarian carcinoma (EOC) is the most common and is associated with the highest mortality, largely due to late detection, aggressiveness and chemoresistance (61, 62, 63). Survivin overexpression has also been observed in human ovarian cancer. Cohen C. et al. reported survivin overexpression in 74% (n = 49) of tissue sections examined, which correlated with poor prognostic parameters, i.e., high grade, histological type and p53 mutation (63). It has also been described that mean survivin mRNA expression levels are higher in ovarian carcinoma than in benign and borderline ovarian tumors and correlate with clinical stage, degree of differentiation and lymph node metastasis (64, 65). The study examined 111 ovarian tissue samples for survivin expression using reverse transcription-polymerase chain reaction, quantified the expression using quantitative real-time PCR and found that survivin was highly expressed in ovarian carcinoma (73%) compared to benign (47%) and borderline (19%) ovarian tumors (*p* < 0.001).

Survivin expression, among other biomarkers, has also been associated with poorer tumor cell differentiation and has been suggested to help assess the aggressiveness of ovarian mucinous, serous and clear cell adenocarcinomas (66). Similarly, studies conducted elsewhere on EOC tissue samples have found a significant correlation between survivin overexpression and increased cell proliferation, malignant potential and poor prognosis (64, 67, 68, 69). As in EC, survivin is involved in several cellular signaling pathways in EOC. It has previously been shown that genetic deletion of survivin gene by a gene-editing technology system using clustered regularly interspaced palindromic repeats and the Cas9 enzyme (CRISPR/Cas9) in orthotopic ovarian cancer mouse models prevented tumor metastasis (70). The underlying mechanisms were further confirmed in another study where ovarian cancer cells resistant to standard chemotherapy were treated with the selective survivin inhibitor MX106. This inhibitor blocked epithelial-to-mesenchymal transition (EMT) by dampening the transforming growth factor-β (TGF-β) signaling pathway, thereby suppressing tumor growth and metastasis (71). EMT is a physiological process in which epithelial cells adopt the migratory and invasive characteristics of mesenchymal cells. When this process is inappropriately activated, often due to changes in the microenvironment or cellular signaling disruptions, it contributes to cancer progression. The three known isoforms of TGF-β in mammals; TGF-β1, β2, and β3 are involved in key physiological processes such as embryonic development, immune responses, proliferation, differentiation, and apoptosis (28).

PTEN alterations in tumors are often due to mutations or deletions. About 20%–30% of ovarian cancers contain mutations in the PTEN gene. While a key step in the development of high-grade serous ovarian cancer (HGSOC) - the most common and lethal subtype - is thought to be TP53 gene mutations that cause protein stabilization in the fallopian tube, accumulating evidence suggests that alterations in PTEN expression may be involved in the early stages of serous ovarian cancer development, similar to endometrioid endometrial cancer (72, 73). Investigation of PTEN expression patterns as a biomarker in 5,400 EOC tissue samples concluded that PTEN loss is a common driver in the development of HGSOC (73). Furthermore, Pradeep S. et al. used mouse models to investigate the role of PTEN in ovarian cancer and observed that simultaneous deletion of PTEN and the liver kinase-B1 (LKB1) induced the development of HGSOC with full penetrance (74). It has also been reported that among ovarian cancer patients, those with PTEN (−)/survivin (+) expression have the worst prognosis (75). The interaction between survivin, PTEN and TP53 in cancer is well described. In addition, the tumor-suppressing effect of caspase-2, a death effector, has been shown to be associated with the silencing of survivin gene transcription (76). Survivin associates with specificity protein-1 (Sp1) to induce chemoresistance in OC patients treated with cisplatin (77). Survivin has also been shown to interact with class III β-tubulin and Sox2 to induce taxane resistance in EOC (78, 79). Several survivin-targeted therapeutic strategies are currently being evaluated in various stages of clinical trials for potential translation into the clinic (Table 2). Among the gynecological cancers, ovarian cancer is relatively less common than endometrial and cervical cancer, but it is still one of the leading causes of gynecological cancer-related mortality.

**TABLE 2:** Survivin-based therapies that have been investigated against various human cancers over the past 2 decades, their mechanisms of action, and their effects on cancer cells.

Compound	Category	Mechanism of action	Reference
Shepherdin	Interruption of survivin-partnerprotein interactions	Interrupts HSP90 interactions with survivin	(99)
AICAR		Prevents binding of survivin to HSP90	(100)
Withanone		Interferes with survivin-Smac interactions	(101)
PZ-6-QN		Inhibits the survivin/Smac interaction and promotes the release of Smac and cytochrome-c	(102)
S12	Disruption of survivin homodimerization	Disrupts survivin dimerization or disrupts a partner protein interaction with survivin monomer	(103)
LQZ-7F		Degrades survivin through a proteasome-dependent pathway, Disrupts microtubule structure	(104)
YM155	Suppression of survivin gene transcription	Selectively inhibits survivin expression at both the protein and mRNA levels	(105)
FL118		Independent inhibition of multiple antiapoptotic gene products (survivin, Mcl-1, XIAP, cIAP2)	(106)
SF002-96–1	Inhibits promoter activity; reduces survivin mRNA levels	Inhibits survivin mRNA and protein expression	(107)
LY2181308	Degradation of survivin mRNA	Reduces survivin mRNA	(108)
Survivin-2B80–88	Survivin-based immunotherapy	Induces anti-tumor immune response	(109)
SurVaxM			(110)

### 2.3 Survivin signaling in cervical cancer

Research has shown that survivin expression in uterine cervical cancer (UCC) is associated with invasiveness, clinical stage, metastasis, chemoresistance and poor prognosis (31, 80, 81, 82). In UCC, survivin has been shown to interact with several molecules involved not only in apoptosis but also in other cell signaling pathways. Overexpression of survivin was significantly associated with poor survival in cervical cancer compared to cancer tissues negative for survivin expression (83). Mechanisms involved in the development of cervical cancer include abnormal cell proliferation and cell cycle deregulation. The p16INK4a, Ki-67, survivin and cyclooxygenase-2 (COX-2) have been described as potential biomarkers involved in the progression of UCC. It has been postulated that survivin may control apoptosis in UCC through interactions with p16INK4a, COX-2 and Ki-67. Indeed, survivin was found to competitively interact with CDK4/p16INK4a upon initiation of cell cycle entry and the correlation of survivin expression levels with p16INK4a, COX-2 and Ki-67 in UCC was significant (84, 85). Furthermore, treatment response in patients with cervical squamous cell carcinoma (CSCC) treated with paclitaxel and carboplatin was independently correlated with grade, Ki67 and survivin expression (86). This suggests that survivin expression may be a marker of prognosis following neoadjuvant therapy in patients with CSCC. This is supported by studies showing that higher levels of survivin expression are associated with treatment resistance in UCC patients treated with cisplatin, paclitaxel and radiotherapy (87, 88, 89). In addition, erythropoietin has been shown to promote survivin expression through activation of STAT3, which negatively affects the sensitivity of cisplatin to cervical cancer cells (90).

Resistance to apoptosis in UCC is often due to loss of TP53 function caused by HPV infection. It has been shown that the use of survivin inhibitors such as sepantronium bromide (YM155) or resveratrol (RSV), a polyphenol with the potential to suppress survivin expression, enhances tumor necrosis factor-related apoptosis-inducing (TRAIL) ligand induced apoptosis and overcome cisplatin resistance in UCC treatment (87). TRAIL induces apoptosis in a p53-independent manner. Survivin has also been suggested as a marker for CIN and high-risk human papillomavirus (HR-HPV) and as a predictor of HPV clearance and disease outcome in cervical cancer (91). Other survivin-related interactions reported in UCC include positive regulation of survivin promoter activity by Sp1 and Sp3, interaction with STAT3 and MDM2, stabilization by HSP90 through physical association, Bcl-2 and KAI1/CD82 proteins, and survivin phosphorylation at Thr34 (92, 93, 94). While extensive work is underway to explore the therapeutic potential of survivin in cancer, attempts are also being made to unravel its diagnostic value. Xue Yan et al. designed survivin-specific molecular beacons (MBs) linked to fluorescein isothiocyanate (FITC) and cyanine-3 (Cy3) dyes and used molecular beacon imaging to investigate the diagnostic potential of survivin. Molecular beacons are single-stranded oligonucleotide probes that form stem-and-loop structures. They found a sensitivity of 61.4% and a specificity of 72.8% (n = 44) for survivin MBs (95). Such findings suggest that survivin MBs may be specific and sensitive probes for the detection of cervical cancer cells. In addition to endometrial, ovarian and cervical cancers, survivin has also been implicated in other gynecological cancers.

### 2.4 Survivin signaling in other gynecological cancers

Data on survivin expression and signaling in vulvar squamous cell carcinoma (VSCC) are scarce, largely due to the rarity of the tumors. Most vulvar cancers are squamous cell carcinomas (90%) and a small percentage (10%) are vulvar melanomas. Squamous cell carcinomas are associated with higher morbidity and mortality because they are highly invasive to adjacent tissues and can metastasize to distant organs (96). There are currently only three published articles on the involvement of survivin in the development of vulvar cancer (29, 30, 97). Gene expression profiling of VSCC by Zhang T et al. found that among the 18 genes associated with the biological characteristics of VSCC, BIRC5 expression was positively and significantly associated with the clinical stage of the tumors (29). In their results, VEGF and MMP1 were also overexpressed, but did not correlate with any of the clinicopathological characteristics examined. Similarly, two other studies found an increase in nuclear survivin immuno-expression from normal epithelium and lichen sclerosus to high grade classic vulvar intraepithelial neoplasia, differentiated vulvar intraepithelial neoplasia and invasive keratinizing squamous cell carcinoma (*p* = 0.0001), suggesting its diagnostic and prognostic potential in VSCC (30, 97). Survivin has been proposed as a molecular target for therapeutic intervention in squamous cell carcinomas (96, 98). Survivin-targeted strategies currently under investigation in gynecological and other tumors include inhibitors of survivin gene transcription, homodimerization, survivin-partner protein interaction, mRNA inhibitors and survivin immunotherapy (Table 2).

Some of the more promising emerging survivin-targeted therapies in cancer include PZ-6-QN, brexpiprazole and SurVaxM. The small molecule PZ-6-QN has been tested in several cancer cell lines, including MCF-7 (breast cancer) and HCT-116 (colon cancer), and has been shown to inhibit the interaction between survivin and the second mitochondrial-derived activator of caspase (Smac) (102). This disruption increases the release of Smac and cytochrome-c from the mitochondria. Smac, a mitochondrial protein, binds to IAPs, neutralizing their inhibitory effect and promoting the activation of caspases, particularly caspase-9 in the cytochrome-c/Apaf-1/caspase-9 pathway, ultimately leading to cell death. Brexpiprazole and SurVaxM were evaluated for their effects on brain tumors cells. Brexpiprazole, a well-established atypical antipsychotic agent, has been shown to increase the sensitivity of glioma cells to the small molecule osimertinib by reducing survivin expression (111). In contrast, SurVaxM is an immunotherapeutic vaccine specifically designed to target survivin-expressing cancer cells. A phase IIa clinical trial demonstrated its significant efficacy in promoting anti-tumors immune responses against brain tumors by generating cytotoxic CD8^+^ and CD4^+^ T-cell responses, resulting in tumors regression and prevention of relapse (112). While these newer survivin-targeted strategies may also prove beneficial for patients with gynecological cancers, there are currently few clinical trials focusing on survivin-based therapies in gynecological cancers (Table 3).

**TABLE 3:** Suvrivin-targeted therapies that are currently in clinical trials to evaluate their anti-tumor activity in the treatment of gynecological cancers.

Compound/Molecule	Treatment/Intervention	Cancer type	Phase	Clinical trial no:
Survivin Peptide	Survivin peptide vaccine	Cervical Cancer	I/II	NCT00108875
Survivin HLA class I peptides	Low-dose oral cyclophosphamide	Ovarian cancer	I	NCT01416038^a^
Survivin HLA class I peptides	Epacadostat and low-dose cytoxan	Ovarian cancer	I/II	NCT02785250
Survivin HLA class I peptides	Low-dose oral cyclophosphamide	Late-stage ovarian/Fallopian tubal cancer	I	NCT03332576
Survivin HLA class I peptides	Low doses of cyclophosphamide and pembrolizumab	Advanced epithelial ovarian/Fallopian tubal cancer	II	NCT03029403
hTERT-mRNA and survivin-peptide		Advanced ovarian cancer	I	NCT01456065
Amplified ovarian cancer stem cell mRNA, hTERT, and survivin	Platinum treatment	Recurrent epithelial ovarian cancer	I/II	NCT01334047^b^

^a^ Completed.

^b^ Terminated.

## 3 Discussion

Survivin is an important multifaceted oncoprotein with tumor-promoting effects in several human cancers. Importantly, survivin plays a major role in cellular proliferation and progression of gynecological tumors. The tumor-promoting effects of survivin in EC and OC are largely dependent on spontaneous mutation of the PTEN and TP53 genes. In UCC, the tumorigenic effects depend on loss of TP53 function due to HPV infection and association with STAT3, MDM2, HSP90 and Sp1/Sp3. Survivin is also associated with resistance to chemoradiation and hormonal therapy in patients with gynecological tumors. Although survivin-based therapies have yet to reach the clinic, survivin inhibitors hold promise for patients with EC, OC, UCC and VSCC, even when they become resistant to standard therapy.

In conclusion, survivin exerts multiple effects in different types of gynecological cancers. Evidence suggests that various survivin-targeted therapeutic strategies such as inhibitors of survivin gene transcription, inhibitors of survivin homodimerization, drugs that disrupt survivin-partner protein interactions, survivin mRNA inhibitors and survivin-based immunotherapy, may be effective either alone or in combination with standard chemotherapy and/or radiotherapy. These approaches have the potential to promote tumor regression, enhance chemo- or radiosensitivity and prevent metastasis. Targeting survivin signaling may therefore improve outcomes for patients with gynecological cancers. However, further research is needed to better understand the role of survivin in the development and metastasis of these cancers, its involvement in resistance to standard therapies, and strategies to overcome such resistance. In particular, clinical trials targeting survivin signaling in gynecological cancer patients are urgently needed.
